# Prospective associations of circulating thrombospondin-2 level with heart failure hospitalization, left ventricular remodeling and diastolic function in type 2 diabetes

**DOI:** 10.1186/s12933-022-01646-x

**Published:** 2022-11-05

**Authors:** CH Lee, MZ Wu, DTW Lui, CHY Fong, QW Ren, SY Yu, MMA Yuen, WS Chow, JY Huang, A Xu, KH Yiu, KSL Lam

**Affiliations:** 1 Department of Medicine, School of Clinical Medicine, University of Hong Kong, Queen Mary Hospital, Pok Fu Lam, Hong Kong; 2 State Key Laboratory of Pharmaceutical Biotechnology, University of Hong Kong, Pok Fu Lam, Hong Kong; 3 Division of CardiologyDepartment of Medicine, the University of Hong Kong-Shenzhen Hospital, Shen Zhen, China

**Keywords:** Thrombospondin-2, Heart failure, Hospitalization for heart failure, Diastolic dysfunction, Diabetes mellitus

## Abstract

**Background:**

Circulating thrombospondin-2 (TSP2) levels were associated with the development of heart failure (HF) in recent studies. However, these studies included only a minority of patients with type 2 diabetes, which is associated with an increased HF risk. As hyperglycemia induces TSP2 expression and its tissue expression increases in type 2 diabetes, we investigated the prospective association of circulating TSP2 with incident HF hospitalization (HHF), and its associations with longitudinal changes of echocardiographic parameters in type 2 diabetes.

**Methods:**

Baseline serum TSP2 levels were measured in 4949 patients with type 2 diabetes to determine its association with incident HHF using multivariable Cox regression analysis. In the echocardiographic study, baseline serum TSP2 levels were measured in another 146 patients with type 2 diabetes but without cardiovascular diseases who underwent detailed transthoracic echocardiography at baseline and after 1 year.

**Results:**

Over a median follow-up of 7.8 years, 330 of 4949 patients (6.7%) developed incident HHF. Baseline serum TSP2 levels were independently associated with the development of HHF (HR 1.31, 95%CI 1.06–1.62, p = 0.014) after adjustments for baseline conventional cardiovascular risk factors, atrial fibrillation, estimated glomerular filtration rate, albuminuria and high-sensitivity C-reactive protein level, use of angiotensin-converting enzyme inhibitors, angiotensin receptor blockers, loop-diuretics, aspirin, insulin, metformin and sodium-glucose co-transporter 2 inhibitors. Moreover, baseline serum TSP2 levels were independently associated with increase in average E/e’ and left atrial volume index (p = 0.04 and < 0.01, respectively).

**Conclusion:**

Serum TSP2 levels were independently associated with both incident HHF and deterioration in diastolic function in type 2 diabetes.

**Trial registration:**

Not Applicable

**Supplementary information:**

The online version contains supplementary material available at 10.1186/s12933-022-01646-x.

## Introduction

Thrombospondin-2 (TSP2) is a matricellular protein that interacts with various ligands such as extracellular matrix (ECM) structural proteins and is implicated in the pathogenesis of cardiovascular diseases (CVD)[1, 2]. Circulating TSP2 levels have been associated with adverse cardiovascular outcomes in patients with heart failure (HF) with preserved and reduced ejection fraction (HFpEF and HFrEF, respectively) [3, 4]. Recently, plasma proteomic profiling had demonstrated that circulating TSP2 level was associated with incident HF in a population-based cohort, as well as the presence of acute HF among individuals attending the emergency department, independent of N-terminal pro-hormone BNP (NT-proBNP) and B-type natriuretic peptide (BNP), respectively [5, 6]. In patients with advanced HF, circulating TSP2 levels decreased significantly from baseline following heart transplantation [5, 6]. However, in all these cohorts, patients with type 2 diabetes only constituted a small proportion, and the prognostic significance of circulating TSP2 levels with regard to HF in type 2 diabetes remains undefined. This is a significant knowledge gap because while patients with type 2 diabetes are at high risk of HF and its adverse outcomes [7, 8], the use of several anti-diabetic agents such as glitazones and sodium glucose co-transporter 2 inhibitors (SGLT2i) has been associated with altered risk of hospitalization for heart failure (HHF) [9, 10]. Most importantly, hyperglycemia induces TSP2 expression and increased tissue expression has been observed in patients with type 2 diabetes [11–13].

In this study with an exclusively diabetic population, we investigated prospectively the association of circulating TSP2 with incident HHF, an outcome that can be significantly reduced with the use of SGLT2i [14–19]. Moreover, the prospective associations between circulating TSP2 and the longitudinal changes of echocardiographic parameters in type 2 diabetes were also evaluated.

## Methods

The study comprised two parts. Part 1 was a prospective cohort study that evaluated the association between baseline circulating TSP2 levels and the development of HHF in type 2 diabetes, whereas Part 2 was an echocardiographic study to investigate whether baseline circulating TSP2 levels were associated with the longitudinal changes in left ventricular (LV) systolic and diastolic functions in type 2 diabetes.

### Study participants

In Part 1, all participants were recruited from the Hong Kong West Diabetes Registry (HKWDR) which consisted of patients with type 2 diabetes regularly followed up at the medical specialist clinics of the Hong Kong West Cluster since 2008. On enrolment to HKWDR, all Chinese patients were invited to participate in a prospective cohort study that aimed to identify the risk factors, including genetic and serum biomarkers, of diabetic complications [20]. In the current study that evaluated the association between serum TSP2 levels and incident HHF, individuals were excluded if they had history of HF, on renal replacement therapy or had received a kidney transplant at baseline. Moreover, only individuals who had follow-up for more than one year after recruitment to HKWDR were included in the analysis to minimize the possibility of reverse causation.

In Part 2, all participants were recruited from the Chinese Diabetic Heart Study (CDHS), which consisted of Chinese patients who had type 2 diabetes but without CVD or severe structural heart disease and were regularly followed up in the medical specialist clinics of Queen Mary Hospital, Hong Kong between July 2011 and August 2014 [21, 22]. All CDHS participants received blood tests and transthoracic echocardiography (ECHO) at baseline to investigate the cardiovascular manifestations and underlying pathophysiology of Chinese patients with type 2 diabetes. These participants were subsequently invited for one follow-up ECHO after at least 1 year.

In both Parts 1 and 2, the study protocols were approved by the Institutional Review Board of the University of Hong Kong / Hospital Authority Hong Kong West Cluster (Ref: UW 07-378 and UW 11–121, respectively). Written informed consent was obtained from all recruited participants prior to any study related procedures.

### Clinical and biochemical assessments

In both Parts 1 and 2, participants attended each study visit after an overnight fast of at least 8 h. At the baseline visit, demographic and anthropometric data including body mass index (BMI), waist circumference (WC), blood pressure (BP) and smoking history were obtained. Fasting blood was collected for baseline evaluation of glycaemic and lipid status, with the remaining blood stored in aliquots at − 70^o^C for assays of biomarkers of diabetic complications. Baseline estimated glomerular filtration rate (eGFR) was calculated using the Chronic Kidney Disease Epidemiology Collaboration (CKD-EPI) equation as described previously [23]. In Part 1, albuminuria status was also assessed using at least two random urine samples on two separate occasions within six months, and categorized according to their urine to albumin creatinine ratio (UACR): <30 mg/g (A1), 30–300 mg/g (A2) and > 300 mg/g (A3) [24]. Serum high sensitivity C-reactive protein (hsCRP) was measured with a high-sensitivity, particle-enhanced immune-turbidimetric assay (Roche Diagnostics, GmbH, Mannheim, Germany).

### Echocardiography assessments

In Part 2 of the study, comprehensive transthoracic ECHO examination was performed using commercially available ECHO machines (Vingmed Vivid E9, General Electric Vingmed Ultrasound, Milwaukee, WI, USA) at baseline and follow-up as described previously [22]. Images were obtained using a 3.5-MHz transducer and digitally stored into three cardiac cycles for analysis by EchoPAC version 112.0 (General Electric Vingmed, Horten, Norway). LV posterior wall thickness and inter-ventricular septal dimension at end-diastole (LVPWd and IVSd, respectively) were measured using a two-dimensional ECHO guided M-mode approach. LV mass was calculated according to the Devereux formula, whereas LV ejection fraction (LVEF) and volumes were assessed using the modified biplane Simpson’s method in both apical two- and four-chamber views. Left atrial volume (LAV) was assessed by single-plane disk summation method in apical four-chamber view. LAV index (LAVi) was determined by LAV divided by the body surface area of the participants. Tissue and pulse-wave Doppler imaging were applied to assess LV diastolic function in apical four-chamber view. Peak trans-mitral flow velocities in early (E-wave) and late diastole (A-wave) were measured to calculate the E/A ratio. The peak velocities of septal and lateral mitral annulus in early diastole (e’) were also determined by tissue Doppler imaging to calculate the average E/e’.

### Measurement of serum TSP2 levels

Serum TSP2 levels were measured with an enzyme-linked immunosorbent assay (ELISA) kit for human TSP-2, which utilized a pair of monoclonal antibodies recognizing distinct sites of human TSP2. (ImmunoDiagnostics Limited, Hong Kong) The assay was highly specific to human TSP2 and did not cross-react with human TSP1, TSP3, TSP4 and TSP5. The lowest detection limit was 0.156 ng/ml. The intra- and inter-assay precision were < 4.6% and < 7.2%, respectively.

### Definitions of clinical variables and outcomes

In our study, dyslipidemia was defined as fasting triglycerides (TG) ≥150 mg/dL, high-density lipoprotein cholesterol (HDL-C) < 40 mg/dL in men and < 50 mg/dL in women, and low-density lipoprotein cholesterol (LDL-C) ≥100 mg/dL, or on lipid-lowering agents. Hypertension was defined as BP ≥140/90 mmHg or the use of anti-hypertensive medications. Although SGLT2i usage has been shown to decrease the risk of HHF in several landmark randomized controlled studies [10], SGLT2i only became available locally after 2015. Therefore, the use of SGLT2i was included as a time-dependent covariate in our prospective evaluation of the association between serum TSP2 levels and incident HHF. SGLT2i usage was defined by the continuous prescription of canagliflozin, dapagliflozin, empagliflozin and ertugliflozin for at least 90 days during the study period from the initiation of medication to the development of outcome or end of observation, whichever was earlier, and classified based on their cumulative daily defined dose (cDDD) as described by the World Health Organization [25]. cDDD reflects the average daily maintenance dose of a drug prescribed for the main indication in an adult. In this study, cDDD was calculated as the sum of dispensed defined daily doses (DDD) of all prescribed SGLT2i during the study period. One DDD of SGLT2i is equivalent to daily use of dapagliflozin 10 mg, empagliflozin 17.5 mg, canagliflozin 200 mg, or ertugliflozin 10 mg.

All outcomes were recorded and verified from the Hong Kong Hospital Authority database or their private practitioners as of 30 June 2020. HHF, the primary outcome of interest in this study, was defined as the first recorded hospitalization with HF as the principle diagnosis coded by a physician based on the ICD-9. The diagnosis were adjudicated and reviewed by two physicians independently, who took into account a constellation of symptoms (shortness of breath, orthopnoea, paroxysmal nocturnal dyspnoea), signs (ankle oedema, basal chest crepitations), chest X-ray or transthoracic ECHO findings if available (pulmonary congestion, suboptimal LVEF) and HF treatment (use of intravenous or increased dose of oral diuretics). Disagreements between the two adjudicating physicians were resolved by a third.

### Statistical analysis

All data were analysed with IBM SPSS Statistics 26.0 (http://www.IBM.com/SPSS) and R version 3.2.3 (http://www.r-project.org). Kolmogorov-Smirnov test was used to test for normality of data. Data that were skewed were either analysed by non-parametric tests, or logarithmically transformed to obtain near normality before analysis. Values were reported as means ± standard deviation (SD), or medians with 25th and 75th percentiles for variables with skewed data, or percentages, as appropriate. Comparisons between groups were analysed by Chi-square test for categorical variables, whereas continuous variables were compared using independent *t* or Mann-Whitney U test as appropriate. Paired *t*-test was performed to compare changes in ECHO parameters between baseline and follow-up. Pearson correlation analysis was conducted to determine the relationship between serum TSP2 levels and other clinical variables. Multivariable Cox regression analysis was performed to evaluate the associations of baseline circulating TSP2 levels with incident HHF. The Cox regression analysis was repeated with use of SGLT2i included as a time-dependent covariate. The variables included in the Cox regression models were those that were statistically or biologically significant in the univariate analysis. The proportional hazards assumption was checked and verified using a global goodness-of fit test proposed by Schoenfeld [26]. The hazard ratio (HR) for circulating TSP2 levels referred to the risk of developing HHF per unit difference in the log-transformed serum TSP2 levels measured in ng/ml. The performance of the clinical model to predict incident HHF, with and without serum TSP2 levels, was examined using time-dependent *c*-statistics, net reclassification index (NRI) and integrated discrimination index (IDI). Linear regression analysis was performed to evaluate the associations of baseline serum TSP2 levels at baseline with longitudinal changes in ECHO parameters. A two-sided *P*-value of < 0.05 was considered significant in all statistical tests.

## Results

### Baseline serum TSP2 levels were higher in patients with type 2 diabetes who developed HHF

In Part 1, a total of 4949 patients with type 2 diabetes were included in the study. At baseline, serum TSP2 levels positively correlated with age, BMI, WC, systolic BP and serum hsCRP levels, and negatively correlated with eGFR levels of the study participants (all p < 0.001). (Supplemental Table S1) Moreover, serum TSP levels were significantly higher in men (p < 0.001), and among participants who had hypertension (p < 0.001), atrial fibrillation (p < 0.001), CVD (p = 0.004) and albuminuria (p < 0.001) than those who did not. (Supplemental Table S2)

Over a median follow-up of 7.8 years, 330 (6.7%) of them developed incident HHF, with a rate of 8.7 per 1000 person-years. Those who developed HHF had significantly longer duration of diabetes (p < 0.001), higher BMI (p = 0.002), WC (p < 0.001), systolic BP (p < 0.001), prevalence of dyslipidemia (p = 0.007), atrial fibrillation (p < 0.001), CVD (p < 0.001) and albuminuria (p < 0.001) at baseline than those who did not. Participants with incident HHF also had significantly higher glycated hemoglobin (HbA1c) and hsCRP, but lower eGFR levels (all p < 0.001) at baseline than those without. Moreover, there were significantly more users of insulin, angiotensin converting enzyme inhibitors (ACEI), angiotensin receptor blockers (ARB), beta-blockers, loop-diuretics, statin and aspirin (all p < 0.001) but less users of metformin (p = 0.021) among participants who had incident HHF than those who did not. Importantly, baseline serum TSP2 levels were significantly higher in participants with incident HHF than those without (Men: 3.70 ng/ml vs. 3.32 ng/ml; women: 4.51 ng/ml vs. 3.71 ng/ml; p < 0.001) (Table 1).

**Table 1:** Baseline characteristics of the participants by incident HHF in Part 1 of the study (N = 4949)

Baseline variables	Incident HHF		p-value
	No	Yes	
Number	4619	330	**--**
Men, %	58.8	57.9	0.737
Age, years	62.2 ± 12.5	70.5 ± 11.6	< 0.001
Smoking			0.479
Non-smoker, %	66.6	64.5	
Ex-smoker, %	23.2	26.1	
Current-smoker, %	10.2	9.4	
Ever-smoker, %	33.4	35.5	0.456
Duration of diabetes, years	11.5 ± 8.5	15.3 ± 9.9	**< 0.001**
Body mass index, kg/m^2^	25.9 ± 4.3	26.6 ± 4.2	**0.002**
Waist circumference, cm			**< 0.001**†
Men	95.0 ± 11.3	90.8 ± 10.6	
Women	85.5 ± 11.2	88.1 ± 10.8	
Systolic BP, mmHg	137.0 ± 20.0	148.0 ± 21.0	**< 0.001**
Diastolic BP, mmHg	75.4 ± 10.1	71.7 ± 10.6	**< 0.001**
Hypertension, %	85.6	97.3	**< 0.001**
Dyslipidemia, %	81.2	95.5	**0.007**
Chronic kidney disease, %	21.7	52.1	**< 0.001**
Atrial fibrillation, %	6.1	16.7	**< 0.001**
Cardiovascular disease, %	31.7	46.1	**< 0.001**
HbA1c, %	7.59 ± 1.36	8.00 ± 1.68	**< 0.001**
HbA1c, mmol/mol	59.4 ± 14.9	63.9 ± 18.4	**< 0.001**
Fasting glucose, mg/dL	59.4 ± 14.9	63.9 ± 18.4	**< 0.001**
Triglyceride*, mg/dL	110.0 (78.8–159.0)	120.0 (88.6–163.0)	**0.002**
HDL-C, mg/dL	47.7 ± 14.2	47.2 ± 14.3	0.538
LDL-C, mg/dL	91.6 ± 31.1	89.3 ± 31.5	0.197
eGFR, mL/min/1.73m^2^	78.5 ± 22.7	59.8 ± 24.3	**< 0.001**
Albuminuria, %	43.4	75.8	**< 0.001**
Use of medications, %			
Insulin	28.8	39.7	**< 0.001**
Metformin	78.5	73.0	**0.021**
Sulfonylureas	48.9	53.6	0.100
Thiazolidinediones	0.9	1.2	0.611
DPP4i	10.0	10.9	0.597
ACEI/ARB	65.1	79.1	**< 0.001**
Beta-blockers	40.1	56.5	**< 0.001**
Loop-diuretics	12.1	29.7	**< 0.001**
Mineralocorticoid antagonist	0.7	1.5	0.094
Statin	49.7	60.9	**0.001**
Fibrates	3.9	3.0	0.439
Aspirin	39.8	56.4	**< 0.001**
hsCRP*, mg/dL	1.06 (0.47–2.63)	1.35 (0.65–3.54)	**< 0.001**
TSP2*, ng/mL			**< 0.001**†
Men	3.32 (2.52–4.54)	3.70 (2.88–3.70)	
Women	3.71 (2.80–5.23)	4.51 (3.45–5.68)	

*Log-transformed before analysis; †Sex-adjusted p-value

TSP2, thrombospondin-2; HHF, hospitalization for heart failure; BP, Blood Pressure; HbA1c, glycated hemoglobin; HDL-C, High-density lipoprotein cholesterol; LDL-C, low-density lipoprotein cholesterol; eGFR, estimated glomerular filtration rate; DPP4i, dipeptidyl peptidase 4 inhibitors; ACEI, angiotensin-converting enzyme inhibitors; ARB, angiotensin receptor blockers; hsCRP, high-sensitivity C-reactive protein

### Baseline serum TSP2 levels were independently associated with incident HHF in type 2 diabetes

In multivariable Cox regression analysis, baseline serum TSP2 levels were independently associated with the development of HHF (HR 1.27, 95%CI 1.03–1.58, p = 0.028), together with age (HR 1.04, 95%CI 1.03–1.06, p < 0.001), duration of diabetes (HR 1.01, 95%CI 1.00–1.03, p = 0.034), BMI (HR 1.04, 95%CI 1.02–1.07, p = 0.003), systolic BP (HR 1.008, 95%CI 1.003–1.010, p = 0.009), HbA1c (HR 1.14, 95%CI 1.05–1.23, p < 0.001), eGFR (HR 0.98, 95%CI 0.98–0.99, p < 0.001), presence of atrial fibrillation (HR 1.96, 95%CI 1.44–2.67, p < 0.001) and albuminuria (HR 2.18, 95%CI 1.65–2.88, p < 0.001), in a model also consisting of sex, smoking status, dyslipidemia, CVD and serum hsCRP levels at baseline (Table 2).

**Table 2:** Multivariable Cox regression analysis showing the associations of serum TSP2 level and other clinical variables with incident HHF (N = 4949)

Baseline variables	HR (95% CI)	p-value	
Men	1.04 (0.80–1.35)	0.795	
Age, years	1.04 (1.03–1.06)	**< 0.001**	
Duration of T2D, years	1.02 (1.00-1.03)	**0.034**	
Ever smoker, %	1.13 (0.86–1.48)	0.375	
BMI kg/m^2^	1.04 (1.02–1.07)	**0.003**	
Systolic BP, mmHg	1.008 (1.003–1.010)	**0.009**	
Dyslipidemia	1.41 (0.82–2.40)	0.211	
Atrial fibrillation	1.96 (1.44–2.67)	**< 0.001**	
CVD	1.25 (0.99–1.58)	0.058	
HbA1c, %	1.14 (1.05–1.23)	**< 0.001**	
eGFR, mL/min/1.73m^2^	0.98 (0.979–0.99)	**< 0.001**	
Albuminuria	2.18 (1.65–2.88)	**< 0.001**	
hsCRP*, mg/dL	1.06 (0.97–1.16)	0.212	
TSP2*, ng/mL	1.27 (1.03–1.58)	**0.028**	

*Log-transformed before analysis

HR, Hazard Ratio; CI, Confidence Interval; TSP2, thrombospondin-2; HHF, hospitalization for heart failure; BMI, body mass index; BP, Blood Pressure; CVD, cardiovascular diseases; HbA1c, glycated hemoglobin; eGFR, estimated glomerular filtration rate; hsCRP, high-sensitivity C-reactive protein

The association between baseline serum TSP2 levels and incident HHF remained significant after adjustments for the use of insulin, metformin, ACEI/ARB, beta-blockers, loop-diuretics and aspirin (HR 1.29, 95%CI 1.04–1.60, p = 0.02), as well as the time-dependent cDDD of SGLT2i (HR 1.31, 95%CI 1.06–1.62, p = 0.014) (Table 3). When study participants were stratified by the presence of CVD at baseline, serum TSP2 levels were significantly associated with incident HHF among those with baseline CVD (p = 0.029) but not in those without. Notably, 9.4% and 5.3% of participants with and without CVD at baseline developed HHF, respectively (Table 3).

**Table 3:** Multivariable Cox regression analysis showing the associations of serum TSP2 level with incident HHF stratified by presence of CVD at baseline (N = 4949)

	N	No. of incident HF (%)	Model 1		Model 2		Model 3	
			Adjusted HR for TSP2 (95% CI)	p-value	Adjusted HR for TSP2 (95% CI)	p-value	Adjusted HR for TSP2 (95% CI)	p-value
All	4949	330 (6.7)	1.27 (1.03–1.58)	**0.028**	1.29 (1.04–1.60)	**0.020**	1.31 (1.06–1.62)	**0.014**
No CVD at baseline	3335	178 (5.3)	1.12 (0.85–1.47)	0.424	1.15 (0.88–1.51)	0.318	1.18 (0.90–1.55)	0.235
CVD at baseline	1614	152 (9.4)	1.50 (1.06–2.13)	**0.022**	1.49 (1.04–2.14)	**0.029**	1.49 (1.04–2.13)	**0.029**

*Log-transformed before analysis

Model 1: Sex, age, duration of diabetes, ever smoker, BMI, systolic BP, dyslipidemia, atrial fibrillation, CVD, HbA1c, eGFR, albuminuria, and serum hsCRP levels at baseline

Model 2: model 1 plus use of insulin, metformin, ACEI/ARB, beta-blockers, loop-diuretics and aspirin at baseline

Model 3: model 2 plus time-dependent cumulative defined daily dose of SGLT2i.

TSP2, thrombospondin 2; HHF, hospitalization for heart failure; HR, hazard ratio; 95%CI, 95% confidence interval; BMI, body mass index, BP, blood pressure; CVD, cardiovascular diseases; HbA1c, glycated hemoglobin; eGFR, estimated glomerular filtration rate; hsCRP, high sensitivity C-reactive protein; ACEI, angiotensin converting enzyme inhibitors; ARB, angiotensin receptor blockers; SGLT2i, sodium glucose co-transporter-2 inhibitors

Since SGLT2i was not locally available until 2015, a sensitivity analysis was further conducted involving only participants who had survived and remained free of HHF in 2015. Among these 4812 participants, 296 developed HHF after 2015. In multivariable Cox regression analysis, baseline serum TSP2 levels remained significantly associated with incident HHF (HR 1.32, 95%CI 1.06–1.64, p = 0.014), together with the use of SGLT2i with cDDD ≥ 180 (HR 0.42, 95%CI 0.20–0.90, p = 0.025) (Supplementary Table S3).

### Discrimination and reclassification performance of the addition of baseline serum TSP2 levels in predicting HHF in type 2 diabetes

The addition of serum TSP2 levels into a clinical model, which consisted of age, sex, duration of diabetes, smoking status, BMI, hypertension, dyslipidemia, atrial fibrillation, CVD, HbA1c, CKD, albuminuria and serum hsCRP level at baseline, led to a significant improvement of *c*-statistics in predicting HHF from 0.79 (95%CI 0.78–0.81) to 0.81 (95%CI 0.79–0.83) (p = 0.003). This was accompanied by improvement in both NRI (0.33, 95%CI 0.26–0.41; p < 0.001) and IDI (0.02, 95%CI 0.01–0.03; p < 0.001) (Table 4),

**Table 4:** Discrimination and reclassification performance of the addition of circulating TSP2 level in predicting HHF in diabetes

	Clinical model without serum TSP2 level	Clinical model with serum TSP2 level	p-value
*c*-statistics (95% CI)	0.79 (0.78–0.81)	0.81 (0.79–0.83)	**0.003**
NRI (95% CI)		0.33 (0.26–0.41)	**< 0.001**
IDI (95% CI)		0.02 (0.01–0.03)	**< 0.001**

Clinical model included age, sex, duration of diabetes, ever-smoker, BMI, hypertension, dyslipidemia, atrial fibrillation, CVD, HbA1c, presence of CKD, albuminuria, and serum hsCRP levels at baseline

TSP2, thrombospondin-2; HHF, hospitalization for heart failure; CI, confidence interval; NRI, net reclassification index; IDI, integrated discrimination index; BMI, body mass index; CVD, cardiovascular disease; HbA1c, glycated hemoglobin; CKD, chronic kidney disease; hsCRP, high-sensitivity C-reactive protein

### Baseline serum TSP2 levels were associated with deterioration in average E/e’ and LAVi in patients with type 2 diabetes but without CVD

In Part 1, the association between baseline serum TSP2 levels and incident HHF seemed to be more readily observed among patients who had type 2 diabetes and CVD. To evaluate whether serum TSP2 levels were still associated with changes in ventricular function in patients with type 2 diabetes but without CVD, a longitudinal echocardiographic analysis was conducted as Part 2 of the study in 146 participants with type 2 diabetes who did not have CVD at baseline. Among them, participants who were in the highest serum TSP2 tertile had significantly higher baseline BMI than those in the lower tertiles (p < 0.01). Otherwise, their baseline clinical characteristics were comparable between the two groups (Supplemental Table S4).

Table 5 summarizes the echocardiographic parameters of these 146 participants at baseline and follow-up after a median interval of 27 months (Range 12–46 months). Compared to participants in the lower baseline serum TSP2 tertiles, those in the highest serum TSP2 tertile had significantly higher LVPWd and LV mass at baseline (both p < 0.05), as well as lower LVEF (p < 0.05), higher average E/e’ (p < 0.01) and LAVi (p < 0.01) at follow-up. Importantly, the increase in average E/e’ and LAVi from baseline were both significantly greater among participants in the highest serum TSP2 tertile than those in the lower tertiles (Both p < 0.05).

**Table 5:** Echocardiographic parameters of the study participants in Part 2 stratified by baseline serum TSP2 tertiles (N = 146)

	First and second serum TSP2 tertiles (N = 98)	Third serum TSP2 tertile (N = 48)	p-value
IVSd (mm)			
Baseline	10.62 ± 1.79	11.05 ± 2.22	0.22
Follow-up	11.19 ± 1.82	11.91 ± 2.18	0.05
Change	0.57 ± 1.28	0.86 ± 1.23	0.20
LVPWd (mm)			
Baseline	9.07 ± 1.10	9.65 ± 1.51	**< 0.05**
Follow-up	9.41 ± 1.40	9.85 ± 1.69	0.10
Change	0.32 ± 1.43	0.19 ± 1.41	0.61
LVM (g)			
Baseline	145.19 ± 37.75	161.46 ± 48.82	**< 0.05**
Follow-up	153.60 ± 40.27	168.53 ± 48.76	0.05
Change	8.41 ± 13.68	7.06 ± 14.07	0.58
LVEF (%)			
Baseline	66.13 ± 3.72	64.99 ± 4.40	0.12
Follow-up	64.38 ± 4.45	62.62 ± 4.63	**< 0.05**
Change	−1.75 ± 5.33	−2.49 ± 5.32	0.45
E/A			
Baseline	0.91 ± 0.24	1.02 ± 0.63	0.13
Follow-up	0.84 ± 0.19	0.91 ± 0.39	0.27
Change	−0.07 ± 0.21	−0.04 ± 0.21	0.51
e’ septal (cm/s)			
Baseline	7.62 ± 1.91	7.54 ± 2.17	0.83
Follow-up	7.12 ± 1.76	6.63 ± 2.07	0.14
Change	−0.47 ± 2.00	−0.92 ± 1.66	0.18
e’ lateral (cm/s)			
Baseline	10.23 ± 2.34	10.13 ± 2.94	0.83
Follow-up	9.51 ± 2.18	8.90 ± 2.54	0.13
Change	−0.77 ± 1.87	−1.23 ± 1.78	0.16
Average E/e’			
Baseline	8.74 ± 2.36	9.53 ± 3.45	0.16
Follow-up	9.36 ± 2.70	11.00 ± 3.75	**< 0.01**
Change	0.57 ± 2.09	1.48 ± 2.35	**< 0.05**
LAVi (ml/m^2^)			
Baseline	30.22 ± 8.23	32.42 ± 10.28	0.19
Follow-up	28.82 ± 7.98	33.78 ± 10.80	**< 0.01**
Change	−1.32 ± 7.35	2.09 ± 8.90	**< 0.05**

TSP2, thrombospondin-2; A wave, trans-mitral late diastolic peak velocity; E wave, trans-mitral early diastolic peak velocity; e’, early diastolic peak velocity of mitral valve at septal or lateral annulus; IVSd, inter-ventricular septal dimension at end-diastole; LAVi, left atrial volume index; LV, left ventricular; LVEF, LV ejection fraction; LVPWd, LV posterior wall thickness at end-diastole

In multivariable linear regression analysis, baseline serum TSP2 levels were positively and independently associated with both changes in average E/e’ (beta 0.75, 95%CI 0.02–1.49, p = 0.04) and LAVi (beta 4.12, 95%CI 1.48–6.77, p < 0.01) (Table 6).

**Table 6:** Univariate and multivariable linear regression analyses showing the associations between baseline characteristics and change in echocardiographic parameters (N = 146)

	Change in Average E/e’					Change in LAVi, ml/m^2^				
	Univariate		Multivariable		Univariate			Multivariable		
	Beta (95%CI)	p-value	Beta (95%CI)	p-value	Beta (95%CI)		p-value	Beta (95%CI)	p-value	
TSP2, ng/ml	0.90 (0.15–1.66)	**0.02**	0.75 (0.02–1.49)	**0.04**	3.41 (0.54–6.28)		**0.02**	4.12 (1.48–6.77)	**< 0.01**	
Baseline Average E/e’	−0.16 (−0.29–−0.03)	**0.02**	−0.24 (−0.38–−0.09)	**< 0.01**						
Baseline LAVi, ml/m^2^					−0.37 (−0.51–−0.23)		**< 0.001**	−0.42 (−0.56–−0.28)	**< 0.001**	
Age, years	0.00 (−0.04–0.04)	0.96	0.02 (−0.03–0.06)	0.46	0.12 (−0.02–0.26)		0.09	0.09 (−0.05–0.22)	0.23	
Men	0.12 (−0.62–0.85)	0.76	−0.03 (−0.72–0.66)	0.93	−0.82 (−3.53–1.89)		0.55	−0.53 (−2.94–1.87)	0.66	
Duration of DM, years*	−0.80 (−2.66–1.07)	0.40			0.17 (−6.40–6.73)		0.96			
BMI, kg/m^2^	0.07 (−0.01–0.15)	0.09	0.05 (−0.03–0.12)	0.20	0.00 (−0.30–0.30)		0.98	−0.03 (−0.30–0.24)	0.84	
Systolic BP, mmHg	0.02 (−0.01–0.04)	0.15			0.07 (−0.01–0.14)		0.08	0.07 (0.00–0.14)	0.05	
Diastolic BP, mmHg	0.03 (−0.01–0.08)	0.12			−0.01 (−0.17–0.16)		0.94			
Smoker	0.12 (−0.72–0.97)	0.77			1.26 (−1.78–4.29)		0.42			
Hypertension	1.06 (0.19–1.92)	**0.02**	1.03 (0.16–1.90)	**0.02**	−0.10 (−3.37–3.18)		0.95			
Dyslipidemia	1.02 (0.21–1.84)	**0.01**	0.86 (0.10–1.63)	**0.03**	1.98 (−1.13–5.09)		0.21			
CKD stage ≥ 3	0.31 (−0.76–1.38)	0.57			3.70 (−0.03–7.43)		0.05	0.71 (−2.86–4.28)	0.70	
HbA1c, %	0.17 (−0.12–0.46)	0.26			1.23 (0.20–2.26)		**0.02**	−0.13 (−1.10–1.36)	0.83	
HbA1c, mmol/mol	0.02 (−0.01–0.04)	0.26			0.11 (0.02–0.21)		**0.02**	0.01 (−0.10–0.12)	0.83	
FG, mg/dL	0.00 (−0.01–0.01)	0.94			0.03 (0.00–0.06)		**0.04**	0.06 (−0.52–0.64)	0.84	
eGFR*, ml/min/1.73m^2^	−0.96 (−3.75–1.83)	0.50			−12.99 (−22.87–−3.10)		**0.01**			
HDL-C, mg/dL	−0.03 (−0.06–0.00)	**0.04**			0.02 (−0.09–0.12)		0.75			
LDL-C, mg/dL	−0.02 (−0.04–−0.01)	**< 0.01**			−0.02 (−0.07–0.04)		0.57			
Triglyceride*, mg/dL	0.00 (0.00–0.00)	0.53			0.00 (−0.01–0.01)		0.92			
Insulin	0.72 (−0.01–1.45)	0.05	0.37 (−0.32–1.05)	0.30	1.47 (−1.24–4.19)		0.29			
Metformin	1.06 (−0.91–3.02)	0.29			−1.48 (−8.58–5.62)		0.68			
Sulfonylureas	−0.10 (−0.83–0.64)	0.80			−1.19 (−3.89–1.51)		0.39			
DPP4i	0.01 (−0.90–0.91)	0.98			−0.33 (−3.69–3.03)		0.85			
ACEI/ARB	0.47 (−0.30–1.22)	0.23			−0.01 (−2.80–2.78)		0.99			
Beta blocker	0.55 (−0.22–1.32)	0.16			1.35 (−1.48–4.19)		0.35			
CCB	0.78 (0.05–1.50)	**0.04**			2.39 (−0.28–5.06)		0.08			
Diuretics	0.88 (−0.37–2.14)	0.17			1.36 (−3.18–5.91)		0.56			
Statin	0.95 (0.22–1.68)	**0.01**			−0.31 (−3.06–2.46)		0.83			

*Log-transformed before data analysis

ACEI, angiotensin-converting enzyme inhibitor; ARB, angiotensin II receptor blocker; BMI, body mass index; CCB, calcium channel blockers; CI, confidence interval; CKD, chronic kidney disease; DBP, diastolic blood pressure; DM, diabetes mellitus; DPP4i, dipeptidyl peptidase-4 inhibitors; E, trans-mitral early diastolic peak velocity; e’, early diastolic peak velocity of mitral valve at septal or lateral annulus; eGFR, estimated glomerular filtration rate; FG, fasting glucose; HbA1c, glycated hemoglobin; HDL-C, High-density lipoprotein cholesterol; LAVi, left atrial volume index; LDL-C, low-density lipoprotein cholesterol; SBP, systolic blood pressure; TSP2, thrombospondin 2

## Discussion

To our knowledge, the current study is the first demonstration of a clinically significant association between circulating TSP2 levels and incident HHF in an exclusively diabetic population, known to be at increased HF risk, and was independent of the use of SGLT2i during the study period. Moreover, using an echocardiographic study, we found that baseline serum TSP2 levels were significantly associated with left ventricular remodeling and the subsequent deterioration of diastolic function in patients with type 2 diabetes but without CVD, highlighting the potential of circulating TSP2 level as a novel biomarker of HF in type 2 diabetes, regardless of the presence of CVD.

Although the association between circulating TSP2 and HF has been demonstrated previously in studies involving community-dwelling individuals and patients across the various stages of HF, only a minority of the participants had type 2 diabetes in all these studies [3–6, 27]. Notably, type 2 diabetes significantly elevates HF risk and is also associated with increased TSP2 tissue expression [7, 11–13]. Therefore, the current study has substantially expanded our understanding of the prognostic importance of circulating TSP2 levels in HF, especially in a study population of high clinical relevance. The use of circulating TSP2 levels, in combination with BNP, was shown to improve the identification of acute HF among individuals attending the emergency department [6]. Here we further demonstrated that the addition of circulating TSP2 levels to conventional clinical variables also significantly improved the prediction of HHF over years in type 2 diabetes, potentially allowing early HF risk stratification and treatment prioritization.

The prospective echocardiographic analysis is another strength of this study. In a recent proteomics study consisting of 999 obese individuals with HFpEF, circulating TSP2 levels were significantly elevated compared with controls and was associated with greater left atrial dilatation, higher LV mass index and E/e’ [27]. Similarly, another recent study demonstrated that circulating TSP2 levels were inversely correlated with LVEF measured at 4 months post-myocardial infarction [28]. Therefore, our findings that circulating TSP2 levels were also associated with indices of LV hypertrophy and subsequent development of diastolic dysfunction in patients without pre-existing CVD or HF at baseline provided further clinical support that TSP2 could possibly be implicated in adverse left ventricular remodeling earlier on, even before the development of clinical HF.

TSP2 is a pro-fibrotic and anti-angiogenic protein that plays a major role in matrix assembly during tissue injury and remodelling processes [29, 30]. In preclinical studies, lack of TSP2 expression in mice after fetal murine cardiomyocyte grafting was shown to promote graft integration, vascularization leading to better graft survival [30]. On the other hand, mice with genetic ablation of TSP2 had difficulty to cope with increased cardiac loading, with augmented matrix metalloproteinase (MMP)-2 and MMP-9 activities which disrupted myocardial matrix integrity, resulting in cardiomyopathy and fatal cardiac rupture [31]. Subsequent studies also demonstrated that the absence of TSP2 predisposed mice to age-related dilated cardiomyopathy, doxorubicin-induced cardiomyopathy and heightened cardiac inflammation in viral myocarditis [24–32, 34]. Interestingly, TSP2 expression in the heart was paradoxically elevated during cardiac injury in both rodents and humans, especially among those that would further progress to HF with worse outcomes [31, 34]. Moreover, TSP2 expression was significantly higher in human hypertrophied hearts that were accompanied by LV systolic dysfunction than those with preserved LVEF [31]. Consistently, in our echocardiographic study, participants in the highest serum TSP2 tertile had significantly greater LV mass and LVPWd at baseline indicating LV hypertrophy. Furthermore, they also experienced significantly more deterioration in average E/e’ and LAVi which were two indices of diastolic dysfunction. Taken together, it is likely that TSP2 activation forms part of an important early-stage molecular program that precedes the development of HF. In other words, the resultant high cardiac TSP2 expression, and possibly its circulating levels in HF, may represent a futile adaptive response that was initially a bodily attempt to overcome HF development. Indeed, recent *in-vitro* studies demonstrated that pro-inflammatory and pro-fibrotic stimuli could increase *THBS2* gene expression, which encodes TSP2, in cardiac fibroblasts [28]. Therefore, in patients with established CVD, this compensatory protective mechanism against cardiac dysfunction might even be more pronounced leading to their higher circulating TSP2 levels. Furthermore, this could also explain the significant reduction in circulating TSP2 levels from baseline following heart transplantation, as demonstrated in recent studies [5, 6], and the observation in the current study that a significant association of serum TSP2 levels with incident HHF was found only amongst those with CVD at baseline.

Our study has a few limitations. First, the observational study design in both parts of our study precludes any inferred causal relationship between high circulating TSP2 levels and incident HF. Secondly, echocardiographic data is not available in Part 1 of our study, and hence rendering it difficult to evaluate the relationship between baseline serum TSP2 levels and longitudinal changes in ECHO parameters in patients who had type 2 diabetes and CVD. Thirdly, serum TSP2 levels were measured only once in all participants and it was therefore impossible to investigate if changes in serum TSP2 levels would also contribute to the development of HHF. Furthermore, external validation of our findings using an independent cohort was not performed. Lastly, neither BNP nor NT-proBNP level was measured in our study. Interestingly, in a preclinical study of hypertensive renin-overexpressing rats, although the cardiac expression of BNP and TSP2 were both up-regulated with pressure overload, it was found that TSP2, but not BNP, more reliably identified those that were prone to develop HF progression [31]. Nonetheless, as ELISA kits for measuring serum TSP2 levels are now commercially available from various sources, further studies, preferably together with the measurements of these established HF markers, are clearly required to validate our current findings, as well as to assess the cost-effectiveness of using this novel biomarker for early HF risk stratification in type 2 diabetes.

## Conclusion

Over the years, reductions in cardiovascular mortality due to coronary heart disease and stroke have been reported especially among older individuals with type 2 diabetes [35]. However, mortality related to HF did not change significantly and remained high. On the other hand, the advent of SGLT2i has introduced a paradigm shift in the management of type 2 diabetes and HF, with several landmark randomized controlled trials showing consistent benefits in reducing the rates of HHF regardless of the presence of CVD and the status of LV function [14–18, 36–40] While we recently demonstrated that circulating TSP2 had the potential to become a novel prognostic marker of liver fibrosis in type 2 diabetes [41], our current findings further proposed that it might also be usefully employed for HF risk stratification in patients with type 2 diabetes.

## Electronic supplementary material

Below is the link to the electronic supplementary material.

Supplementary Material 1. Supplemental Table S1 Pearson correlation analysis of serum TSP2 level with clinical variables at baseline. Supplemental Table S2 Serum TSP2 level and baseline clinical characteristics at baseline. Supplementary Table S3 Sensitivity analysis showing the association between baseline circulating TSP2 levels and incident HF hospitalization in participants who survived and remained free of outcome events in 2015 (N = 4812). Supplementary Table S4 Baseline characteristics of the participants by serum TSP2 levels in Part 2 of the study (N = 146).

## Data Availability

The datasets generated and/or analysed during the current study are available from the corresponding author on reasonable request.

## Abbreviations

A wave: Peak trans-mitral flow velocities in late diastole
ACEI: Angiotensin converting enzyme inhibitors
ARB: Angiotensin II receptor blockers
BMI: Body mass index
BNP: B-type natriuretic peptide
BP: Blood pressure
BW: Body weight
cDDD: Cumulative daily defined dose
CDHS: Chinese Diabetic Heart Study
CI: Confidence interval
CKD-EPI: Chronic kidney disease epidemiology collaboration
CVD: Cardiovascular diseases
DDD: Daily defined dose
E’ Peak: velocities of septal and lateral mitral annulus in early diastole
ECHO: Echocardiography
ECM: Extracellular matrix
eGFR: Estimated glomerular filtration rate
E-wave: Peak trans-mitral flow velocities in early diastole
HbA1c: Glycated haemoglobin
HDL-C: High-density lipoprotein cholesterol
HF: Heart failure
HFpEF: Heart failure with preserved ejection fraction
HHF: Hospitalization for heart failure
HKWDR: Hong Kong West Diabetes Registry
HR: Hazard ratio
hsCRP: High sensitivity C-reactive protein
IDI: Integrated discrimination index
IVSd: Inter-ventricular septal dimension at end-diastole
LAV: Left atrial volume
LAVi: Left atrial volume index
LDL-C: Low-density lipoprotein cholesterol
LV: Left ventricular
LVEF: Left ventricular ejection fraction
LVPWd: Left ventricular posterior wall thickness at end-diastole
MMP: Matrix metalloproteinase
NRI: Net reclassification index
NTproBNP: N-terminal pro-hormone BNP
SD: Standard deviation
SGLT2i: Sodium glucose co-transporter 2 inhibitors
TG: Triglyceride
TSP2: Thrombospondin-2
UACR: Urine to albumin creatinine ratio
WC: Waist circumference
