# Supplementary material for: Prospective associations of circulating thrombospondin-2 level with heart failure hospitalization, left ventricular remodeling and diastolic function in type 2 diabetes
Source: Cardiovasc Diabetol. 2022 Nov 5;21:231. doi: 10.1186/s12933-022-01646-x (PMC9637303; doi:10.1186/s12933-022-01646-x)
Supplement: Supplementary file 1 — Supplementary Material 1. Supplemental Table S1 Pearson correlation analysis of serum TSP2 level with clinical variables at baseline. Supplemental Table S2 Serum TSP2 level and baseline clinical characteristics at baseline. Supplementary Table S3 Sensitivity analysis showing the association between baseline circulating TSP2 levels and incident HF hospitalization in participants who survived and remained free of outcome events in 2015 (N = 4812). Supplementary Table S4 Baseline characteristics of the participants by serum TSP2 levels in Part 2 of the study (N = 146). [file 12933_2022_1646_MOESM1_ESM.pdf]

Supplemental Table S1 Pearson correlation analysis of serum TSP2 level with clinical variables at baseline

	Sex-adjusted r	p-value
Age, years	0.12	**<0.001**
BMI, kg/m^2	0.14	**<0.001**
WC, cm	0.09	**<0.001**
Duration of diabetes, years	-0.03	0.071
Systolic BP, mmHg	0.14	**<0.001**
Diastolic BP, mmHg	-0.01	0.485
Fasting glucose, mg/dL	0.03	0.052
HbA1c, %	-0.03	0.086
eGFR, ml/min/1.73m^2	-0.17	**<0.001**
hsCRP*, mg/L	0.16	**<0.001**

*Log-transformed before analysis

TSP2, thrombospondin-2; BMI, body mass index; WC, waist circumference; BP, blood pressure; HbA1c, glycated hemoglobin; eGFR, estimated glomerular filtration rate; hsCRP, high sensitivity C-reactive protein.

Supplemental Table S2 Serum TSP2 level and baseline clinical characteristics at baseline

	TSP2* level, ng/mL		p-value
	No	Yes	
Sex (Men)	3.35 (2.55-4.60)	3.74 (2.83-5.26)	**<0.001**
Hypertension	2.97 (2.27-3.96)	3.58 (2.72-5.00)	**<0.001**
Dyslipidemia	3.47 (2.56-4.77)	3.51 (2.66-4.89)	0.863
Atrial Fibrillation	3.47 (2.63-4.79)	4.29 (3.08-5.71)	**<0.001**
CVD	3.43 (2.59-4.87)	3.66 (2.78-4.89)	**0.004**
Albuminuria	3.23 (2.46-4.35)	3.95 (2.93-5.44)	**<0.001**

*Log-transformed before analysis
TSP2, thrombospondin-2; CVD, cardiovascular diseases.

Supplementary Table S3 Sensitivity analysis showing the association between baseline circulating TSP2 levels and incident HF hospitalization in participants who survived and remained free of outcome events in 2015 (N=4812)

	Adjusted HR (95% CI)	p-value
Men	1.05 (0.79-1.40)	0.711
Age, years	1.05 (1.03-1.07)	**<0.001**
BMI, kg/m^2	1.03 (1.00-1.07)	0.062
Ever-smoker	1.05 (0.79-1.40)	0.723
Duration of diabetes, years	1.01 (1.00-1.03)	0.163
Systolic BP, mmHg	1.005 (1.00-1.01)	0.110
Dyslipidaemia	1.33 (0.76-2.33)	0.315
Atrial fibrillation	1.90 (1.36-2.65)	**<0.001**
eGFR, mL/min/1.73m^2	0.99 (0.980-0.995)	**0.001**
CVD	0.81 (0.59-1.12)	0.207
Albuminuria	2.01 (1.50-2.69)	**<0.001**
HbA1c, %	1.15 (1.05-1.26)	**0.003**
Use of metformin	1.39 (1.01-1.92)	**0.045**
Use of insulin	1.11 (0.84-1.48)	0.461
Use of ACEI/ARB	1.60 (1.18-2.16)	**0.002**
Use of aspirin	1.17 (0.87-1.58)	0.302
Use of loop-diuretics	1.96 (1.42-2.71)	**<0.001**
Use of beta-blockers	1.13 (0.88-1.45)	0.351
Use of SGLT2i†		
No SGLT2i	Referent	
cDDD <180	1.83 (0.74-4.55)	0.193
cDDD ≥180	0.42 (0.20-0.90)	**0.025**
hsCRP*, mg/L	1.07 (0.97-1.18)	0.158
TSP2*, ng/mL	1.32 (1.06-1.64)	**0.014**

*Log-transformed before analysis; †Time-dependent covariate

TSP2, thrombospondin-2; HR, hazard ratio; 95%CI, 95% confidence interval; BMI, body mass index; BP, blood pressure; eGFR, estimated glomerular filtration rate; CVD, cardiovascular diseases; HbA1c, glycated hemoglobin; ACEI, angiotensin-converting enzyme inhibitors; ARB, angiotensin receptor blockers; SGLT2i, sodium glucose co-transporter 2 inhibitors; cDDD, cumulative defined daily dose; hsCRP, high-sensitivity C-reactive protein.

Supplementary Table S4 Baseline characteristics of the participants by serum TSP2 levels in Part 2 of the study (N=146)

	Total (n=146)	First and second tertiles (N=98)	Third tertile (N=48)	p-value
TSP2, ng/ml				
Men		TSP2<2.32	TSP2≥2.32	
Women		TSP2<2.92	TSP2≥2.92	
Age, years	61±10	61±9	61±10	0.85
Men, %	52.7	53.1	52.1	0.91
Duration of diabetes, years	16 (11-22)	17 (11-23)	15 (10-20)	0.42
BMI, kg/m^2	26±5	26±4	28±5	**<0.01**
Systolic BP, mmHg	138±18	136±18	140±19	0.28
Diastolic BP, mmHg	80±9	80±8	79±10	0.85
Smoker, %	24.7	21.4	31.3	0.20
Hypertension, %	78.1	73.5	87.5	0.05
Dyslipidemia, %	73.3	72.4	75.0	0.74
CKD stage ≥3, %	13.7	11.2	18.8	0.21
HbA1c, %	7.72±1.27	7.65±1.23	7.88±1.34	0.30
HbA1c, mmol/mol	60.91±13.86	60.07±13.45	62.62±14.65	0.30
Fasting glucose, mg/dL	150.3±51.4	147.5±49.1	156.0±55.8	0.35
eGFR, ml/min/1.73m^2	88.9 (73.4-98.3)	90.4 (75.8-98.5)	83.1 (66.8-97.7)	0.26
HDL-C, mg/dL	49.9±13.0	50.3±12.0	48.9±14.8	0.54
LDL-C, mg/dL	92.0±25.5	94.8±24.1	86.4±27.7	0.07
Triglyceride, mg/dL	106.3 (75.3-150.6)	97.4 (75.3-141.7)	110.7 (73.1-183.8)	0.22
Insulin, %	43.8	40.8	50.0	0.29
Metformin, %	96.6	96.9	95.8	1.00
Sulfonylureas, %	52.1	56.1	43.8	0.16
DPP4i, %	21.2	18.4	27.1	0.23
ACEI/ARB, %	63.7	59.2	72.9	0.11

Beta blocker, %	35.6	30.6	45.8	0.07
CCB, %	50.0	45.9	58.3	0.16
Diuretics, %	9.6	10.2	8.3	0.95
Statin, %	58.9	59.2	58.3	0.92

ACEI, angiotensin-converting enzyme inhibitors; ARB, angiotensin II receptor blockers; BMI, body mass index; BP, blood pressure; CCB, calcium channel blockers; CKD, chronic kidney disease; DBP, diastolic blood pressure; DPP4i, dipeptidyl peptidase 4 inhibitors; eGFR, estimated glomerular filtration rate; HbA1c, glycated hemoglobin; HDL-C, high-density lipoprotein cholesterol; LDL-C, low-density lipoprotein cholesterol; TSP2, thrombospondin 2.
